# Unsolicited Patient Complaints Following the 21st Century Cures Act Information-Blocking Rule

**DOI:** 10.1001/jamahealthforum.2023.3244

**Published:** 2023-09-29

**Authors:** Robert J. Dambrino, Henry J. Domenico, John A. Graves, Melinda J. B. Buntin, William Martinez, S. Trent Rosenbloom, William O. Cooper

**Affiliations:** 1 Department of Neurological Surgery, Vanderbilt University Medical Center, Nashville, Tennessee; 2 Department of Health Policy, Vanderbilt University Medical Center, Nashville, Tennessee; 3 Department of Biostatistics, Vanderbilt University Medical Center, Nashville, Tennessee; 4 Center for Patient and Professional Advocacy, Vanderbilt University Medical Center, Nashville, Tennessee; 5 Bloomberg School of Public Health, Johns Hopkins University, Baltimore, Maryland; 6 Carey Business School, Johns Hopkins University, Baltimore, Maryland; 7 Department of Medicine, Vanderbilt University Medical Center, Nashville, Tennessee; 8 Department of Pediatrics, Vanderbilt University Medical Center, Nashville, Tennessee; 9 Department of Biomedical Informatics, Vanderbilt University Medical Center, Nashville, Tennessee

## Abstract

**Importance:**

The 21st Century Cures Act includes an information-blocking rule (IBR) that requires health systems to provide patients with immediate access to their health information in the electronic medical record upon request. Patients accessing their health information before they receive an explanation from their health care team may experience confusion and may be more likely to share unsolicited patient complaints (UPCs) with their health care organization.

**Objective:**

To evaluate the quantity of UPCs about physicians before and after IBR implementation and to identify themes in UPCs that may identify patient confusion, fear, or anger related to the release of information.

**Design, Setting, and Participants:**

This retrospective cohort study was conducted with an interrupted time-series analysis of UPCs spanning January 1, 2020, to June 30, 2022. The data were obtained from a single academic medical center, Vanderbilt University Medical Center, at which the IBR was implemented on January 20, 2021. Data analysis was performed from January 11 to July 15, 2023.

**Exposure:**

Implementation of the IBR on January 20, 2021.

**Main Outcomes and Measures:**

The primary outcome was the monthly rate of UPCs before and after IBR implementation. A qualitative analysis was performed for UPCs received after IBR implementation. The Wilcoxon rank-sum test was used to compare monthly complaints between the pre- and post-IBR groups. The Pearson χ^2^ test was used to compare proportions of complaints by UPC category between time periods.

**Results:**

The medical center received 8495 UPCs during the study period: 3022 over 12 months before and 5473 over 18 months after institutional IBR implementation. There was no difference in the monthly proportions of UPCs per 1000 patient encounters before (median, 0.81 [IQR, 0.75-0.88]) and after (median, 0.83 [IQR, 0.77-0.89]) IBR implementation (difference in medians, −0.02 [95% CI, −0.12 to 0.07]; *P* =.86). Segmented regression analysis revealed no difference in monthly UPCs (β [SE], 0.03 [0.09]; *P* =.72).

**Conclusions and Relevance:**

In this cohort study, implementation of the Cures Act IBR was not associated with an increase in monthly rates of UPCs. These findings suggest that review of UPCs identified as IBR-specific complaints may allow clinicians and organizations to prepare patients that their test and procedure results may be available before clinicians are able to review them and respond.

## Key Points

**Question:**

Does the 21st Century Cures Act information-blocking rule (IBR) affect unsolicited patient complaints (UPCs)?

**Findings:**

In this cohort study conducted at a single institution, 8495 UPCs were identified in an interrupted time-series analysis. There was no difference in the rate of UPCs after IBR implementation in January 2021; however, complaint themes related to the policy were identified in the qualitative review of UPCs.

**Meaning:**

These findings suggest that opportunities remain for clinicians to prepare patients for the possibility that test and procedure results may be available to them before clinicians are able to review them and respond.

## Teaser

This cohort study quantifies and evaluates themes present in unsolicited patient complaints at a single institution before and after implementation of the 21st Century Cures Act information-blocking rule in 2021.

## Introduction

The 21st Century Cures Act (hereinafter, the Cures Act), which became law on December 13, 2016, and entered its first compliance phase on April 5, 2021, was designed to increase patient access to their health information and to facilitate innovation in the pharmaceutical and medical device arena.^1,2^ The Cures Act included the information-blocking rule (IBR), which was intended to remove barriers that limit timely patient access to their own health information. The IBR included provisions that required physicians and health care organizations to provide access to all clinical information to patients upon their request and without delay.^3^ Under the new rule, patients can access all designated electronic health information, including consultation notes, history and physical examinations, radiology reports, laboratory and pathology reports, discharge summaries, and progress and procedures notes.^3^

Although the IBR was intended to improve access to health data, it is possible that such immediate access may cause patient confusion, anxiety, and unhappiness in certain circumstances.^4,5,6,7,8^ For example, a patient may experience distress if they read a new anatomic pathology report revealing incident cancer before their health care professional is able to explain the results. Patient distress can lead to increased numbers of unsolicited patient complaints (UPCs).^9^ These complaints provide important information about individual and organizational risk of adverse patient outcomes and malpractice claims.^10,11,12^ In addition, UPCs can be reliably coded to identify types of complaints related to care and treatment, unclear communication, concern for patients and families, accessibility, safety of the environment, and billing concerns.^13^ We are not aware of any study to date that has evaluated the association of IBR implementation with UPCs overall and by type.

We aimed to evaluate the number of UPCs about physicians and the content of these complaints before and after implementation of the Cures Act IBR at a large academic medical center. We hypothesized that the numbers of UPCs would increase as a result of IBR implementation. Our secondary hypothesis was that certain types of complaints would reflect patient concerns related to confusion, anxiety, and clinician availability to answer patient questions related to release of information.

## Methods

### Design and Setting

We conducted this retrospective cohort study with interrupted time-series analysis (1) to measure rates of UPCs per patient visit over time and (2) to determine whether there was a change coincident with implementation of the Cures Act IBR. This study was performed at Vanderbilt University Medical Center (VUMC), a large US academic medical center with 1709 beds. On January 20, 2021, the VUMC patient portal, My Health at Vanderbilt (MHAV), was modified to provide immediate access to all designated electronic health information, in advance of the IBR April 2021 compliance date. Prior to January 20, 2021, the institutional policy was to release radiology reports 3 business days after results were available, to release pathology reports 14 calendar days after available, and to never release clinical notes electronically.^14^ The study period spanned from January 1, 2020, to June 30, 2022, which included 385 days prior to VUMC implementation of immediate access and ended 525 days later. Immediate access to medical records included providing all clinical data (including progress notes, pathology reports, and radiology reports) immediately through MHAV. During the study period, there were 1 115 905 unique patients at VUMC, of whom 653 455 (58.6%) were enrolled in MHAV. Of those enrolled, 447 124 (68.4%) were active on MHAV during the study period. Users of MHAV were not linked to specific UPCs based on prior publication data.^11^ All data used for the study were deidentified by a data manager (employed by VUMC) not involved in the conduct of the research prior to investigator receipt of the deidentified research data set. The VUMC Institutional Review Board reviewed the study, determined that it did not qualify as human participants research per CFR §46.102(e)(1), and waived informed consent. The study followed the Strengthening the Reporting of Observational Studies in Epidemiology (STROBE) reporting guideline.

### Study Population

The study population included patients treated at the study site, and the unit of measurement was UPCs per 1000 encounters per month. Unsolicited patient complaints from resident physicians and fellows were not included in the analysis.

### Data Sources

The Vanderbilt Center for Patient and Professional Advocacy (CPPA) works with hospitals and medical groups across the US to identify affiliated physicians with increased numbers of UPCs. The CPPA maintains an electronic database housing patient complaint data for more than 60 000 US physicians credentialed at participating health care institutions. The CPPA identifies high-risk physicians as those whose volume of complaints shows them to be outliers locally and nationally among their peers.^10,15^ Unsolicited patient complaints from Vanderbilt physicians in the CPPA database were used as the source of data for this study.

Data on patient encounters were obtained from the VUMC Enterprise Data Warehouse. Counts included the total number of outpatient encounters per month.

### Outcomes

The primary study outcome was monthly rates of UPCs from any setting. These rates were calculated by dividing the total number of UPCs by outpatient encounters and are expressed as UPCs per 1000 encounters.

### Qualitative Analysis

A validated coding algorithm for UPC types was used to categorize complaint types before and after IBR implementation, with coders trained to approximately 90% reliability.^13^ A subset of 1822 post-IBR complaints coded for UPCs pertaining to the categories of communication, documentation, treatment, and diagnosis (CDTD) were evaluated to assess complaint themes attributable to the IBR. To identify UPCs associated with the patient online health portal, a review of the complaints was performed using the following terms: *MHAV*, *My Health*, *My Health at Vanderbilt*, *portal*, and *app*. Examples from this group of UPCs were independently examined by 2 authors (R.J.D. and W.O.C.) to identify patient concerns potentially related to the IBR. Consensus was used to determine final groupings.

### Statistical Analysis

The monthly rate of UPCs among physicians per 1000 patient encounters was compared before and after IBR implementation. Monthly complaint rates were compared using a Wilcoxon rank-sum test and are reported using medians (IQRs). Differences in group medians with bootstrapped 95% CIs using 1000 bootstrap replicates are reported. Proportions of complaints by UPC category were compared between time periods using the Pearson χ^2^ test. A cohort study with interrupted time-series analysis was implemented to analyze the total and monthly rates of any category of complaints about physicians as well as complaints related to CDTD categories before and after January 20, 2021, the date VUMC implemented the changes to comply with the Cures Act IBR.^16^ The time-series analysis used a segmented regression model with parameters for changes in mean and slope in the post-IBR period compared with the pre-IBR period. The month of implementation (January 2021) included both pre- and post-IBR exposures. This month was treated as a washout period and was excluded from the analysis. To assess whether UPC rates were affected by COVID-19 during the pre-IBR period, a sensitivity analysis was performed using monthly UPCs during 2019 as the pre-IBR period (eFigure in Supplement 1). Two-sided *P* < .05 was considered statistically significant. All statistical analyses were performed with R, version 4.2.3 (R Foundation for Statistical Computing). Data analysis was performed from January 11 to July 15, 2023.

## Results

### UPCs Before and After IBR Implementation

We identified 8495 UPCs during the study period: 3022 over 12 months before and 5473 over 18 months after institutional implementation of the IBR. The median monthly rate of complaints per 1000 encounters was 0.81 (IQR, 0.75-0.88) in the pre-IBR period compared with 0.83 (IQR, 0.77-0.89) in the post-IBR period (difference in medians, −0.02 [95% CI, −0.12 to 0.07]; *P* =.86; Table 1). For the CDTD categories, the median monthly rate of UPCs per 1000 patient encounters was 0.35 (IQR, 0.32-0.38) in the pre-IBR group and 0.33 (IQR, 0.32-0.35) in the post-IBR group (difference in medians, 0.02 [95% CI, −0.02 to 0.05]; *P* =.34). The proportion of total UPCs related to physician accessibility and availability was higher in the post-IBR period (764 [14.0%]) than in the pre-IBR period (375 [12.4%]) (difference in proportions, 1.6% [95% CI, 0.03% to 3.1%]; *P* =.08; Table 2). Compared with the pre-IBR period, the proportion of complaints related to care and treatment (1534 [50.8%] vs 2698 [49.3%]; difference in proportions, −1.5% [95% CI, −3.7% to 0.8%]; *P* =.46) and communication (757 [25.0%] vs 1288 [23.5%]; difference in proportions, −1.5% [95% CI, −3.5% to 0.4%]; *P* =.22) was slightly lower in the post-IBR period. However, no association was observed between time period and overall complaint classification by UPC category (Table 2).

**Table 1.** Complaint Comparison Before and After the 21st Century Cures Act IBR Institutional Compliance Date (January 20, 2021)^a^

	Before IBR (n = 12 mo)	After IBR (n = 17 mo)	Overall (n = 29 mo)
Total unsolicited patient complaints about attending physicians, No. (95% CI)	250.5 (225.2-279.0)	332.0 (310.0-354.0)	302.0 (258.0-335.0)
Specific complaints about physicians related to CDTD, No. (95% CI)	116.0 (98.0-121.5)	132.0 (126.0-142.0)	126.0 (116.0-135.0)
Total patient encounters, No. (95% CI)	333 797.0 (303 162.5-340 356.2)	397 834.0 (383 771.0-409 239.0)	372 595.0 (333 983.0-400 084.0)
Complaints about physicians, per 1000 encounters (95% CI)	0.81 (0.75-0.88)	0.83 (0.77-0.89)	0.83 (0.77-0.89)
Complaints about physicians related to CDTD, per 1000 encounters (95% CI)	0.35 (0.32-0.38)	0.33 (0.32-0.35)	0.33 (0.32-0.37)

Abbreviations: CDTD, communication, documentation, treatment, or diagnosis; IBR, information-blocking rule.

^a^ Complaint rates were compared using a Wilcoxon rank-sum test.

**Table 2.** Proportion of UPCs by Type Before and After Implementation of the 21st Century Cures Act IBR

Complaint type	No. (%) of UPCs		*P* value^a^
	Before IBR (n = 3022)	After IBR (n = 5473)	
Care and treatment	1534 (50.8)	2698 (49.3)	.09
Communication	757 (25.0)	1288 (23.5)	
Accessibility	375 (12.4)	764 (14.0)	
Concern for patient and/or family	234 (7.7)	472 (8.6)	
Money or payment	115 (3.8)	239 (4.4)	
Safety of environment	7 (<0.1)	12 (<0.1)	

Abbreviations: IBR, information-blocking rule; UPC, unsolicited patient complaint.

^a^ Proportions of complaints by UPC category were compared between time periods using a Pearson χ^2^ test.

**Figure 1.**
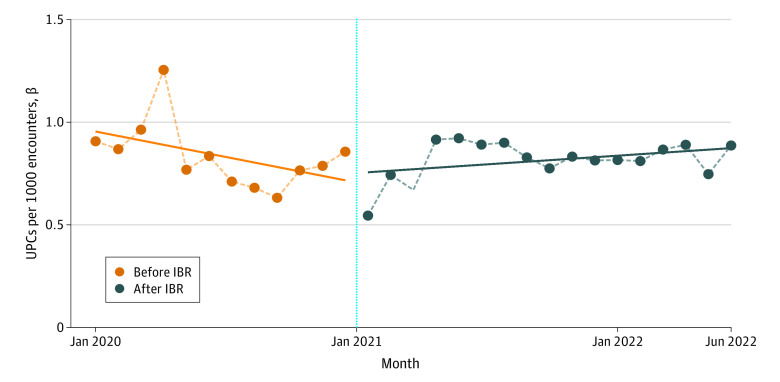
Interrupted Time Series of Unsolicited Patient Complaints (UPCs) per 1000 Encounters Before and After Implementation of the 21st Century Cures Act Information-Blocking Rule (IBR)

**Figure 2.**
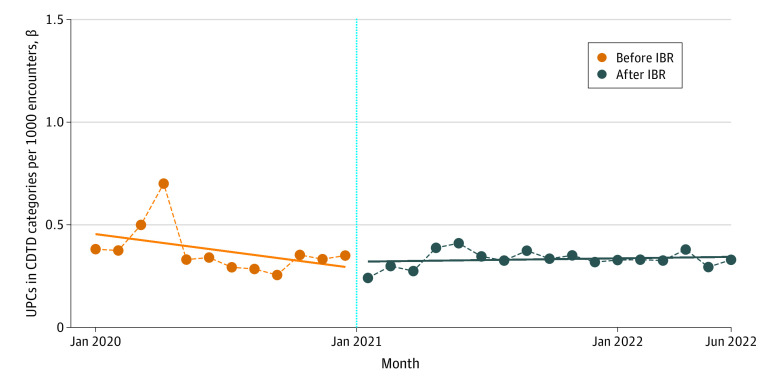
Interrupted Time Series of Unsolicited Patient Complaints (UPCs) in Communication, Documentation, Treatment, and Diagnosis (CDTD) Categories per 1000 Encounters Before and After Implementation of the 21st Century Cures Act Information-Blocking Rule (IBR)

Segmented regression analysis for all monthly UPCs per 1000 patient encounters revealed no statistically significant change in average monthly rates of UPCs from the pre-IBR and post-IBR periods (β [SE], 0.03 [0.09]; *P* =.72) (Figure 1). Statistical models using 2019 as the pre-IBR period were not materially different from models using 2020 (eFigure in Supplement 1). For the CDTD subgroup of UPCs, segmented regression analysis also demonstrated no statistically significant difference in average monthly rates of CDTD UPCs in the pre-IBR and post-IBR groups (β [SE], 0.02 [0.06]; *P* = .67) (Figure 2).

### IBR-Related Complaints

A total of 1822 complaints included references to the MHAV portal and underwent subsequent review. Examples from the post-IBR period included the following: concerns with diagnostic results from radiology or pathology reports, disagreements with documentation in the medical record, anxiety related to an unexplained medical finding, complaints about medical team communication, and unexpected findings found in the medical record (Table 3).

**Table 3.** UPCs Related to the 21st Century Cures Act

UPC type referencing online patient portal	UPC
Diagnostic results from radiology and pathology reports	“I am very upset and concerned that my MRI results mentioned ‘cannot rule out lymphoma.’”
Documentation in the medical record	“I was given an EEG and told to follow up with neurology. The emergency room doctor said my EEG was normal. The portal says my EEG was abnormal. What is happening?”
Anxiety related to unexplained medical finding	“I spent all weekend terrified after reading some terrifying information … I could not reach my doctor to discuss.”
Medical team communication	“My visit note mentions a diagnosis that my physician never examined me or tested me for.”
Unexpected findings found in medical record	“When I logged back on to My Health at Vanderbilt, there were test results listed that no one told me they were performing. Why was this done? I want these test results purged from my medical records.”

Abbreviations: EEG, electroencephalogram; MRI, magnetic resonance imaging; UPC, unsolicited patient complaint.

Many of the complaint themes arose in the setting of the patient having access to a report (eg, a radiology or pathology report) before they had an opportunity to speak with their physician. For example, a complaint about a magnetic resonance imaging (MRI) report was as follows: “patient waiting for Dr. *** to call her with results from MRI … very upset and concerned that her MRI results mention lymphoma.” Another patient complained, “I spent all weekend terrified after reading some terrifying information … I could not reach my doctor to discuss.”

There were also several reports in which patients expressed surprise at what they found in their medical record or stated that they may have had a test they were unaware was performed. One patient stated, “I was given an EEG [electroencephalogram] and told to follow up with neurology. The emergency room doctors said my EEG was normal. The portal says my EEG was abnormal. What is happening?” Another patient noted, “I logged back on to My Health … and several, test results were listed that no one told me would be performed… I respectfully request that … an explanation be given for why these tests were performed. I also request … that the test results be purged from my medical record.”

## Discussion

In this large, single-institution study, we observed that there was no difference in the monthly rate of overall UPCs and CDTD-related UPCs among physicians before and after implementation of the Cures Act IBR when adjusted for numbers of patient encounters. Although there were specific complaints that were directly related to the IBR, the rate of overall complaints did not increase.

A major concern among different physician groups around IBR implementation has been increased patient anxiety and anger toward clinicians as a direct result of patient misunderstanding of components of the medical record.^7,17^ Furthermore, release of all of the patient’s medical information in real time could reveal inaccurate information about their health condition, which could lead to UPCs. Unsolicited patient complaints are a known validated estimator of malpractice activity; however, because of the typical lag time between events and malpractice claim filing and resolution, claims data are not currently available to corroborate or refute the notion that increased IBR-related UPCs would estimate subsequent malpractice claims.^10^ Given our findings, the lack of difference in UPCs between the pre- and post-IBR groups suggests that a large increase in malpractice claims related to medical record information release through the IBR should not be expected.

The Cures Act requires immediate electronic availability upon patient requests for all test results, medication lists, and clinical notes. The Cures Act does not require health care institutions to push unrequested designated electronic health information to patient portals. However, given the real-world constraints of existing commercially available electronic health record systems and patient portals, many health care systems are providing immediate, real-time access via their patient portals as the only feasible solution for accommodating the IBR.^18^ In a recent study by Steitz et al,^19^ immediate access to all health results resulted in a substantial difference in patients accessing them before their physician did. In addition, the number of daily messages sent to physicians nearly doubled from a median of 78 to 146 after the transition to IBR compliance.^19^ This increase in patient messaging suggests that the IBR may increase some patient concerns that may not rise to the level of a UPC. Although follow-up research by that team indicated that patients prefer to continue having immediate access to their electronic health information 95% of the time, the risk of increased physician work remains.^19^

### Unintended Consequences of the IBR

Although our study did not reveal substantial differences in the monthly proportion of UPCs per patient encounter, the types of complaints seen after IBR implementation were specific to results found in the medical record. In our qualitative analysis of post-IBR UPCs, themes were identified regarding diagnostic results from radiology and pathology reports, documentation in the medical record, anxiety related to unexplained medical findings, medical team communication, and unexpected findings in the medical record. By empowering patients to have ownership of their health care, having electronic health information immediately available could improve communication with their practitioners.^20^

### Clinical and Policy Implications

These findings offer insight for clinicians to consider when they are ordering tests or performing procedures, particularly in an effort to temper patient expectations. The concept of open notes has been widely implemented across the US and other countries for more than a decade and has been well studied and characterized.^21^ Survey data suggest that patients, particularly those receiving oncology care, have more trust and higher satisfaction with open notes.^22,23,24,25,26^ A recent survey study with respondents from 4 academic medical centers revealed that 96% of patients preferred immediate release of and access to online test results even if their health care practitioner had not yet reviewed them.^19^ However, without adequately preparing patients and adjusting clinical systems, the increased messaging seen in the immediate release of electronic health information could increase the workload of clinicians and their clinical staff.^19^ To mitigate this increased messaging, clinicians and health care organizations can prepare patients for the possibility that results may be posted at the same time or even before the clinician has a chance to review them.^27^ Practices could build in processes to ensure that patient questions are answered in a timely manner.

A best practices guideline would be helpful for both the practitioner and the patient to anticipate some of these anxiety-provoking reports that may lead to IBR-specific UPCs.^28^ For example, a practice called *precounseling* involves communicating to the patient that they should expect results to populate in their online portal and to consider waiting to view them until their follow-up appointment or phone conversation. Also, having a mechanism within a practice to follow up with patients on potentially worrisome results could help to reassure patients that communication will occur if there is a concern. Consideration of patient preferences when receiving protected health information (PHI) could help facilitate better health care communication. For example, patients may choose to see only abnormal laboratory values or may opt out of viewing any PHI. Also, given that patients did not have access to clinical notes in the pre-IBR period, access could have provided rationale for the tests ordered and decreased confusion among patients.

Many of the changes mentioned would require clarification in the law as written and could be included in the recent IBR changes proposed by the US Department of Health and Human Services Office of the National Coordinator for Health Information Technology.^29^ Some best practices for PHI receipt or opt-out options may need to be specifically addressed in the Cures Act to avoid be considered information blocking. In addition, information blocking as defined in the Cures Act could still be taking place and not yet have any precedent set for prosecution. The definition of the term *developer* is important because developers participating in information blocking are subject to penalties, whereas practitioners are not. These topics provide an opportunity for clarification in updates to the Cures Act.

### Limitations

Our study has several limitations. This was a single-institution study representing an academic health system, which may not be representative of other US academic practices or nonacademic health systems. Our institution implemented MyChart (Epic) in 2017, so these findings may only translate to those who use the Epic electronic medical record system. Although the Cures Act IBR requires immediate access upon patient request, it does not necessarily require immediate, real-time access to an online portal as implemented at our study site. Other health care entities may have different implementation dates and different strategies that could further affect the generalizability of these findings. A multi-institutional study would strengthen the findings of this study and should be pursued in the future. In addition, our study only examined complaints that were tied to physicians, because our goal was to estimate malpractice activity with UPCs. Some complaints not aimed at physicians but still related to immediate access to PHI in MHAV may have occurred and were not captured in our analysis.

Of the 1 115 905 unique patients at VUMC, 653 455 (58.6%) were enrolled in MHAV during the study period. Of those patients enrolled in MHAV, only 447 124 (68.4%) were active. Thus, many patients may not be aware of the IBR or may not have experienced any change in their health care experience as a result. The institutional transition for Cures Act compliance occurred during the height of the COVID-19 pandemic and could have affected patient experiences, which may have resulted in UPCs, including hospital volume and access to care.^30^ To account for this, we performed a sensitivity analysis using the prior year (2019) data and observed no difference in the interrupted time series.

## Conclusions

In this cohort study with interrupted time-series analysis, the Cures Act IBR was not associated with a change in the monthly rate of UPCs at a large academic medical center. A qualitative review of the complaints suggests that there are unintended consequences of complex medical information being immediately available to patients. Further study of the effects of this legislative mandate with multi-institutional data and a longer time horizon may be helpful for further understanding of this law’s effect on UPCs.

## Notes

Supplement 1. **eFigure.** Interrupted Time Series With 2019 Data: Patient Complaints per 1000

Supplement 2.
Data Sharing Statement
