# Supplementary material for: Unsolicited Patient Complaints Following the 21st Century Cures Act Information-Blocking Rule
Source: JAMA Health Forum. 2023 Sep 29;4(9):e233244. doi: 10.1001/jamahealthforum.2023.3244 (PMC10543134; doi:10.1001/jamahealthforum.2023.3244)
Supplement: Supplement 1. — eFigure. Interrupted Time Series With 2019 Data: Patient Complaints per 1000 [file jamahealthforum-e233244-s001.pdf]

# Supplementary Online Content

Dambrino RJ IV, Domenico HJ, Graves JA, et al. Unsolicited patient complaints following the 21st Century Cures Act information-blocking rule. *JAMA Health Forum*. 2023;4(9):e233244. doi:10.1001/jamahealthforum.2023.3244

**eFigure.** Interrupted Time Series With 2019 Data: Patient Complaints per 1000

This supplementary material has been provided by the authors to give readers additional information about their work.

© 2023 Dambrino RJ IV et al. *JAMA Health Forum*.

**eFigure.** Interrupted Time Series With 2019 Data: Patient Complaints per 1000

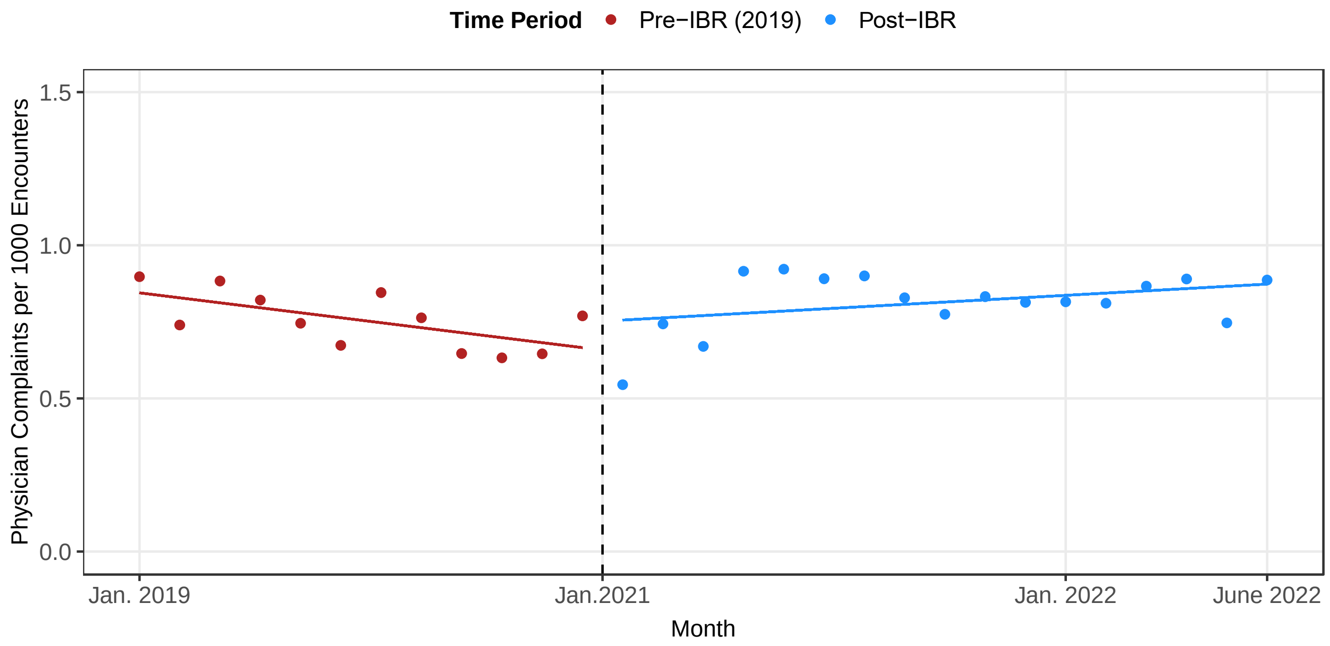


| | β | S.E. | P value |
|---|---|---|---|
| **Intercept** | 0.86 | 0.05 | <0.0001 |
| **Observation Month** | -0.02 | 0.01 | 0.03 |
| **Period = Post-IBR** | 0.08 | 0.06 | 0.20 |
| **Post-IBR months** | 0.02 | 0.01 | 0.01 |

© 2023 Dambrino RJ IV et al. *JAMA Health Forum*.
