# Supplementary material for: Unsolicited Patient Complaints Following the 21st Century Cures Act Information-Blocking Rule
Source: JAMA Health Forum. 2023 Sep 29;4(9):e233244. doi: 10.1001/jamahealthforum.2023.3244 (PMC10543134; doi:10.1001/jamahealthforum.2023.3244)
Supplement: Supplement 2. — Data Sharing Statement [file jamahealthforum-e233244-s002.pdf]

Data Sharing Statement

Dambrino, IV. Unsolicited Patient Complaints Following the 21st Century Cures Act Information-Blocking Rule. *JAMA Health Forum*. Published September 29, 2023. doi:10.1001/jamahealthforum.2023.3244

Data
Data available: No
